# The Role of Nutraceuticals and Probiotics in Addition to Lifestyle Intervention in the Management of Childhood Obesity—Part 2: Comorbidities

**DOI:** 10.3390/nu17091487

**Published:** 2025-04-28

**Authors:** Maria Elisabeth Street, Federica Casadei, Erika Rita Di Bari, Francesca Ferraboschi, Anna Giuseppina Montani, Maria Concetta Mele, Anna-Mariia Shulhai, Susanna Esposito

**Affiliations:** 1 Department of Medicine and Surgery, University of Parma, 43121 Parma, Italy; federica.casadei@unipr.it (F.C.); erikarita.dibari@unipr.it (E.R.D.B.); francesca.ferraboschi@unipr.it (F.F.); annagiuseppina.montani@unipr.it (A.G.M.); annashulhai@gmail.com (A.-M.S.); susannamariaroberta.esposito@unipr.it (S.E.); 2 Unit of Paediatrics, P. Barilla Children’s Hospital, University Hospital of Parma, 43121 Parma, Italy; maria.mele@ao.pr.it

**Keywords:** obesity, nutraceuticals, probiotics, hypertension, bone mineral density, polycystic ovary syndrome, mental health

## Abstract

Pediatric obesity is associated with a wide range of comorbidities beyond metabolic changes, affecting cardiovascular, endocrine, reproductive, musculoskeletal systems, and also mental health. Hypertension, commonly observed in children with obesity, increases the risk of long-term cardiovascular disease. Polycystic ovary syndrome (PCOS) presents another significant endo-reproductive challenge that often develops during adolescence in females, leading to further comorbidities in adulthood. Additionally, excess adiposity can negatively impact bone health by modifying bone metabolism and increasing fracture risk. Obesity is also strongly linked to mental health disorders, including depression, anxiety, and low self-esteem, which can further exacerbate unhealthy lifestyle behaviors and disorders. Given the limitations and poor adherence of traditional treatment strategies, nutraceuticals have emerged as potential complementary therapies due to their bioactive properties. Various compounds have demonstrated antihypertensive, insulin-sensitizing, and anti-inflammatory effects, while others support bone metabolism and promote mental well-being. Herewith, we discuss the role of nutraceuticals in managing hypertension, PCOS, bone health, and mental health issues in individuals with obesity, evaluating their mechanisms of action and clinical relevance. Integrating nutraceutical compounds with dietary and lifestyle interventions may improve treatment outcomes and prevent obesity-related comorbidities. Further, we emphasize the need for further large-scale clinical studies, especially in pediatric patients.

## 1. Introduction

One of the most emerging global health challenges now is the rising incidence of childhood obesity. According to recent data, more than 8% of children in the world are obese [1]. Obesity is a complex, multifactorial condition that is associated with many adverse health outcomes and significantly increases the risk of various comorbidities. Among the most recurrent complications in obese subjects are [2,3,4] PCOS [5,6], bone fragility [7], and the development of mental and emotional health disorders [8], which should be considered by physicians.

Obesity and weight-gain during childhood are linked to obesity-related health problems and chronic disorders in adulthood [9]. Therefore, physicians are seeking additional complementary strategies for obesity management beyond the traditional dietary interventions, lifestyle modifications, and pharmacological therapy, that often have limitations and poor adherence in both children and adults [10]. There is growing interest in the role of nutraceutical compounds as alternatives to the management of obesity and obesity-related comorbidities [11]. Nutraceutical bioactive compounds are obtained from natural sources having nutritional and pharmacological properties [11].

Nutraceuticals have been investigated for their potential to modulate key physiological pathways involved in obesity-related complications [10,11,12,13]. The biological benefits rely mostly on polyphenols, inositols, antioxidants, fibers, and unsaturated fatty acids, and probiotics as they may improve endothelial function, reduce oxidative stress, inflammation, and modulate the renin-angiotensin-aldosterone system [13,14,15]. Moreover, these compounds may contribute to the improvement of insulin sensitivity, hormonal balance [16], and the reduction of the adverse effects of excess adiposity on bone mineral density [17]. Recent evidence also suggests that, due to the anti-inflammatory and neuroprotective effects, they can play a role in reducing symptoms of depression and anxiety in overweight/obese individuals [18,19].

In this narrative review, we describe nutraceuticals and bioactive compounds that have been shown to have effects on single obesity-related complications and comorbidities, such as hypertension, PCOS, loss of bone mineral density, and mental disorders. To the best of our knowledge, this is the first comprehensive review to address these comorbidities collectively in the context of pediatric obesity, spanning from childhood to adulthood. Based on the current scientific evidence, the benefits and limitations of single nutraceutical compounds are reported, highlighting those with available scientific support for clinical practice guidance and improvement of overall health in obese individuals.

## 2. Materials and Methods

Studies were identified through a comprehensive search across MEDLINE, PubMed, and Google Scholar. Relevant studies highlighting the impact of nutraceutical supplementation on obesity/overweight and related complications were selected using the following keywords: “pediatric”, and/or “child”, and/or “children”, and/or “childhood”, and/or “adolescent”, and “obesity”, and/or “overweight”, and/or “pediatric obesity”, and/or “hypertension”, and/or “blood pressure”, and/or “metabolic syndrome”, and/or “insulin resistance”, and “polycystic ovary syndrome”, and “bone health”, and/or “bone”, and/or “osteoporosis”, and “mental health”, and/or “depression”, and/or “anxiety” AND “nutraceuticals”, and/or “dietary supplements”, and/or “anti-obesity agents”, and/or “vitamins”, and/or “vitamin D”, and/or “antioxidants”, and/or “phytosterols”, and/or “flavonoids”, and/or “polyphenols”, and/or “probiotics”, and/or “prebiotic”, and/or “PUFA”, and/or “omega 3”, and/or “omega 6”, and/or “olive oil, and/or “green tea”, and/or “inositols”, and/or “alpha lipoic acid”. These keywords were differently matched to obtain the search strings. Systematic reviews, meta-analyses, randomized controlled clinical trials, controlled clinical trials, original articles, and case series were taken into consideration. All considered articles were published in English. The search was performed from 1993 to 2024. Whenever studies were missing for the age group 0–18 yr, we considered studies in adults with findings that could be extended potentially to childhood. Both in vivo and in vitro studies were additionally considered for the single compounds of greater interest. A thorough analysis and subsequent synthesis of the literature gathered from the search were conducted and are presented below. In part 2, we separately considered evidence on the effects of nutraceuticals in obese individuals with hypertension, PCOS, osteoporosis, and mental health disorders as comorbidities.

## 3. Hypertension and Evidence for the Use of Nutraceuticals

The increase in childhood obesity has been paralleled by an increased prevalence of childhood hypertension [20]. In fact, observations have shown that excess weight in childhood and adolescence is the leading cause of hypertension [21]. Hypertension occurs in 5% of overweight and 15.3% of obese children, compared to just 1.9% in children with normal weight. There are no notable differences in prevalence based on gender or whether children live in urban or rural environments [22,23]. The pathophysiology of hypertension in obese children is complex and not yet fully understood, with central obesity playing a significant role, in addition to uric acid levels and insulin resistance [21,24]. Elevated levels of leptin and insulin presented in obese subjects, along with endothelial dysfunction and oxidative stress, create a chronic proinflammatory state that increases sympathetic activity, causes vasoconstriction, and leads to sodium and fluid retention via the renin-angiotensin-aldosterone system (RAAS), ultimately resulting in elevated BP [25]. Moreover, low levels of adipocytokine adiponectin (AD), known for its anti-inflammatory and insulin-sensitizing effects, have been associated with childhood obesity and hypertension [26].

The crucial aspect of managing obesity-related hypertension is lifestyle changes that mainly include a calorie-restricted diet, sodium reduction, and increased physical activity [27]. The American Academy of Pediatrics and the USA National Heart, Lung, and Blood Institute primarily recommend overweight subjects to follow the Dietary Approaches to Stop Hypertension (DASH) guidelines [23]. When lifestyle changes are ineffective or in cases of symptomatic hypertension, pharmacological intervention may be needed [28,29] starting with the nutraceutical compounds.

Considering any use of nutraceuticals, studies suggest that omega-3 polyunsaturated fatty acids (*PUFAs*), especially docosahexaenoic acid (*DHA*) and eicosapentaenoic acid (*EPA*), may reduce cardiovascular mortality and help manage hypertension. Their anti-hypertensive effects derive from improved endothelial function, decreased vascular resistance, increased nitric oxide production, reduced inflammation, and regulated cardiac output [30]. Omega-3 *PUFAs* are converted to linolenic acid, an essential intermediate in the production of *EPA* and *DHA*. Both *EPA* and *DHA* can serve as precursors for the synthesis of molecules with modulatory activity on inflammation and blood pressure (BP). Since humans have low efficiency in converting linolenic acid to *DHA* and *EPA*, an imbalance in omega-3 *PUFAs* may affect peripheral vascular resistance and BP [31]. In relation to this, research using animal models has demonstrated that diets rich in omega-3 *PUFA* can protect against hypertension. A recent meta-analysis confirmed beneficial effects of omega-3 *PUFAs* on reduction and BP regulation in adults [32,33]. Furthermore, there is increasing evidence showing the benefits of omega-3 fatty acid supplementation in decreasing systolic and diastolic BP in overweight children. A study involving school-age children with metabolic syndrome demonstrated that a daily intake of 2.4 g of omega-3 fatty acids for one month resulted in a significant decrease in both systolic and diastolic blood pressure [34]. Another randomized study in overweight adolescent boys reported that a 16-week fish oil diet (1.5 g/day of long-chain omega-3 fatty acids) reduced systolic and diastolic BP by 3.8 mmHg and 2.6 mmHg, respectively [35].

In a study involving sixty obese children, the dietary intake of a combination of 130 mg of *DHA* and 25 mg of *EPA* with antioxidant vitamins (200 µg vitamin A, 1.25 µg vitamin D, 2.5 mg vitamin E, and 30 mg vitamin C) significantly improved both systolic and diastolic BP values over three months [36]. A further study conducted in fifth- and sixth-grade obese children reported that the dietary intake of 100 to 350 mg omega-3 PUFAs decreased BP in a dose-dependent manner compared with controls [37]. However, there is currently no large population-based study to support these findings.

Childhood obesity has been associated with low concentrations of circulating *vitamin D* [38]. *Vitamin D* plays a critical role in regulating cardiometabolic risk factors, such as glucose homeostasis and insulin secretion [39]. Studies have shown that children with low levels of vitamin D often have higher BP, and *vitamin D* deficiency is associated with arterial hypertension [40]. Additionally, vitamin D may reduce the activity of the renin-angiotensin-aldosterone system (RAAS) and decrease the BP by reducing angiotensin II level and inhibiting renin activity [41]. Moreover, insufficient vitamin D levels can lead to inefficient regulation of endothelial nitric oxide synthase (NOS) transcription, which may impair endothelium-dependent vascular relaxation and enhance vascular contractile activity [42]. Among adults, a wealth of observational data has demonstrated a relationship between low serum levels of *vitamin D* and hypertension [43], although there was no evidence of a clinically significant reduction in BP because of *vitamin D* supplementation [44]. Some 2022 prospective cross-sectional studies have shown that *vitamin D* deficiency was three times more prevalent in obese children compared to lean children, with this trend being more noticeable in winter months. Hypertension was found only in obese children, and those with *vitamin D* deficiency had the highest BP levels. Interestingly, seasonal variations in *vitamin D* were a key predictor of changes in systolic BP [42], which has also been confirmed in further studies [38,45]. A study involving obese children aged 10 to 18 years examined the effects of three different doses of oral *vitamin D*: 600 IU, 1000 IU, and 2000 IU, respectively, taken three times a day for six months. The results showed a reduction in BP with minimal differences among the groups [39]. However, these results are controversial, as a recent meta-analysis reported no effect of *vitamin D* supplementation on BP in children and adolescents [40].

In conclusion, omega-3 fatty acid supplements may represent a tolerable and safe option for children suffering from obesity-induced hypertension. However, further well-structured studies are necessary to define an appropriate dose and verify the beneficial effects of *PUFA* supplementation on BP in the pediatric patients. Current evidence also suggests that vitamin D deficiency is a risk factor for arterial hypertension in obese children. However, it is not fully understood how it is involved in the pathogenesis of pediatric hypertension, and future research on this topic is needed. Optimizing vitamin D serum levels may serve as a primary preventive role for long-term cardiovascular health but further studies are necessary to clarify the possible benefit of vitamin D supplementation.

The main nutraceuticals studied in children with obesity-related hypertension are summarized in Table 1.

## 4. Bone Health and Evidence for the Use of Nutraceutical Compounds

Childhood and adolescence are characterized by rapid and significant longitudinal bone growth with substantial, areal bone expansion, and bone mineral accrual [46]. Bone development is determined by genetic factors [47] and environmental factors, both non-modifiable and modifiable, such as diet, physical activity, and hormonal stimuli. Emerging evidence supports an interaction between adipose tissue and bones [48]. Young obese subjects present increased differentiation of mesenchymal stem cells into adipocytes at the expense of osteoblasts. In case of decreased physical activity, the balance between osteoblasts and osteoclasts through the RANK/RANKL and osteoprotegerin pathways is altered and on a high-fat diet, calcium (Ca^2+^) bioavailability is reduced [49]. Moreover, obesity correlates with an inflammatory framework that negatively impacts bone health. Increased leptin and decreased adiponectin that characterize obesity are negatively associated with bone mass [50,51]. Certainly, improving lifestyle according to the guidelines for the treatment of obesity is the first helpful step to prevent bone health loss.

Studies concerning the use of nutraceuticals to prevent bone loss are currently scarce for both children and adults. A few in vitro studies, however, have provided interesting findings. *Polyphenol sweet cherries*, for example, are able to inhibit osteoclastogenesis and reduce pro-osteoclastogenic cytokines, without affecting cell viability in peripheral blood mononuclear cell cultures from school-age obese children [52].

In vivo, *green tea polyphenol* (GTP) supplementation for 4 months in female rats on high-fat or calorie-restricted diets showed a positive effect on bone microstructure and strength, with increased femoral mass and strength, trabecular thickness and number, cortical thickness of tibia, decreased trabecular separation, formation rate, and eroded surface at the proximal tibia [53]. GTP supplementation reduced serum insulin-like growth factor (IGF-I) and leptin concentrations, but had no effect on serum adiponectin level in rats [54].

*Extra virgin olive oil* and the Brazilian diet increased bone mineral density and serum calcium, but reduced parathyroid hormone (PTH) levels [55]. Interestingly, researchers have shown that conjugated *linoleic acid* supplementation, that is omega-6 fatty acid, for three months instead had no effect on bone mineral content and density, possibly because of the short intervention period in overweight/obese [56], and further investigations are required.

*Vitamin D* is involved in children’s bone development [57]. Recent investigation showed induced bone dysplasia by altering triglyceride synthesis in *vitamin D* deficient subjects [57]. However, there are conflicting results on the effects of *vitamin D* supplementation [58]. Even though vitamin D supplemetantion is associated with bone mineral density [58], there is no evidence that *vitamin D* supplementation (1200–2000 IU/day) has beneficial effects on bone status in obese children during weight-loss programs [58,59].

In conclusion, there is a lack of population-based studies on the effects of nutraceuticals on bone mineral density and overall bone health. Some in vitro and in vivo studies have shown positive effects of *polyphenols* on bone mineral density, indicating that further clinical inquiries are needed. Research involving adults has demonstrated that omega-6 fatty acids, *vitamin D* supplementation, and extra virgin olive oil can be safe and beneficial when combined with weight-loss programs. However, as some results are controversial, further research is required to confirm the long-term effects of these interventions on bone health in overweight and obese individuals.

## 5. Polycystic Ovary Syndrome and Evidence for the Use of Nutraceutical Compounds

Polycystic ovary syndrome (PCOS) is an endocrine condition that typically emerges during adolescence, causing infertility and metabolic disorders. PCOS is characterized by irregular menstrual periods with anovulation, hyperandrogenism, and polycystic ovaries [60,61]. Although obesity does not serve as a necessary or sufficient determinant of PCOS, it has been observed that individuals with PCOS often present visceral adiposity, and obese subjects with PCOS manifest a more pronounced phenotype than their normal-weight counterparts [5]. Obesity can exacerbate the metabolic changes already present in PCOS subjects, by worsening insulin resistance and fostering hyperinsulinemia which then contributes to adipogenesis [61,62]. Additionally, obesity contributes to sensitizing thecal cells to luteinizing hormone (LH), leading to increased ovarian androgen production [61].

The implementation of comprehensive lifestyle intervention is well known to have positive effects on women with PCOS [62]. In a clinical trial involving obese adolescent girls with PCOS, the combination of lifestyle interventions (including nutritional education, exercise, and behavioral therapy) led to weight-loss, lower levels of androgen and LH, and a significant increase in sex hormone-binding globulin (SHBG). Alongside, girls experienced improved blood pressure, glucose metabolism, insulin sensitivity, and blood lipids [63].

The use of 15 g of *essential amino acids* (10% histidine, 11% isoleucine, 32% leucine, 16% lysine, 10% phenylalanine, 10% threonine, and 11% valine) per day for 28 days in PCOS adolescents with obesity improved aspartate aminotransferase (AST) and triglyceride levels, and reduced liver fat content. However, it did not change the body mass index, androgen levels, and SHBG levels in these subjects [64].

Recent research has established a link between gut dysbiosis and metabolic and reproductive derangements in women with PCOS. *Probiotic* supplementation stands as a promising therapeutic option for addressing these manifestations [65].

A six-month *probiotic* supplementation with six different strains of *Lactobacilli*, *Bifidobacterium bifidum*, and fructo-oligosaccharides combined with dietary and lifestyle modifications improved menstrual regularity, testosterone, and insulin levels in PCOS women, along with a significant weight-loss [66]. Another study established that probiotics containing four *Lactobacillus* strains in combination with cyproterone acetate therapy reduced inflammation markers and weight in PCOS women [67]. Moreover, *Synbiotic* supplementation in combination with lifestyle modifications in obese women with PCOS enhanced anthropometric parameters and reduced free testosterone levels [68].

Growing evidence supports the beneficial properties of dietary *polyphenols*. Studies have demonstrated their role in mitigating metabolic changes associated with obesity, primarily through their anti-inflammatory and antioxidant properties [69,70]. Furthermore, several studies have evaluated the impact of polyphenols in obese or overweight women diagnosed with PCOS [70,71,72,73,74,75].

For example, drinking *green tea* (500 mg/twice a day) for 12 weeks improved body weight and reduced fasting insulin and testosterone in obese and overweight women with PCOS [71]. However, no studies are available to prove any efficacy in adolescents.

*Curcumin* supplementation (500 mg/day) for 12 weeks showed beneficial effects on body mass index (BMI), glucose, and lipid metabolism compared to placebo [72]. A further study showed that hyperandrogenism was improved after *curcumin* (500 mg/three times a day) supplementation for 12 weeks [73]. A randomized clinical trial confirmed the efficacy of lower dose curcumin supplementation (93.34 mg) for 8 weeks in reducing body weight, BMI, fat mass, and in improving insulin sensitivity. However, no significant improvement in hormone levels was reported [74].

Daily consumption of the polyphenol quercetin, 1000 mg for 12 weeks, was shown to be effective in reducing insulin resistance, insulin levels, testosterone, and LH serum levels [75] in PCOS women.

Studies reported reduced hyperandrogenism and improved menstrual cycle following *resveratrol* intake (1000–1500 mg/day) for 3 months [76,77].

However, there were no significant effects on weight-loss, metabolic profile, and androgen levels.

A randomized clinical trial evaluated the efficacy of a weekly intake of a *pomegranate juice* drink supplemented with *probiotics* on glucose homeostasis, showing improved insulin sensitivity, a reduction in weight and in testosterone levels [78]. These findings are supported by another study evaluating the effects of *pomegranate juice* (45 mL/day) after 8 weeks [79].

High-dose *carnitine*, *L-arginine*, and *N-acetylcysteine* were shown to counteract hyperinsulinemia and metabolic changes associated with PCOS [80,81]. Based on this initial evidence, a clinical trial was conducted in overweight/obese adult women with PCOS who received daily supplementation with *carnitine* (250 mg), *L-arginine* (500 mg), and *N-acetylcysteine* (50 mg) for 24 weeks. Following treatment, a reduction in insulin levels and Homeostatic Model Assessment for Insulin Resistance (HOMA-IR) index, improved lipid profile, decreased triglycerides and total cholesterol, and increased HDL-C were described [82].

*Berberine* supplementation (500 mg twice/day) for 6 months improved visceral adiposity, insulin resistance, and lipid profile, reduced androgen levels, and improved menstrual regularity in PCOS women [83].

*Inositols* belong to the B-vitamin group and have beneficial effects on the metabolic and hormonal changes associated with PCOS [84]. *Inositols* exist in several stereoisomeric forms, the main ones being *Myo-inositol* (*MYO*) and *D chiro-inositol* (*DCI*). These are naturally found in plasma in a 40:1 ratio and both have insulin-sensitizing properties. The administration of a *MYO* and *DCI* combination proved to be effective in improving metabolic and hormonal parameters, reducing insulin, HOMA-IR, testosterone, and LH levels, as well as enhancing ovulatory function and increasing SHBG levels in women with PCOS [85].

*Inositol* intake (100 mg/twice a day) for 16 weeks, in addition to folic acid, B vitamins, iron, zinc, and iodide, affected ovarian function in women with PCOS by obtaining a higher frequency of ovulation and increased estradiol levels, suggestive of improved follicular maturation. Additionally, this combined treatment led to significant weight-loss, reduced leptin levels, and increased HDL-C. However, this therapy did not show any metabolic benefits in patients with severe obesity (BMI > 37 kg/m^2^) [86].

The combination of *MYO* and *DCI* supplementation in a 40:1 ratio (550 mg MYO + 13.8 mg DCI, twice daily) has been shown to be more effective than *MYO* monotherapy in improving metabolic and ovarian function in overweight women with PCOS [87]. Additionally, in obese women, combined *MYO* and *DCI* therapy has been found to enhance metabolic and hormonal parameters, while improving insulin sensitivity and ovulatory function [88]. Furthermore, the intake of *MYO* + *DCI* supplementation, in addition to diet, has proven to be more effective in improving anthropometric measurements and menstrual regularity than MYO monotherapy combined with diet or diet alone [89].

Alpha-lipoic acid (*ALA*) is a powerful natural antioxidant that has been shown to have beneficial effects in the treatment of PCOS due to its ability to improve insulin sensitivity [90] and reduce oxidative stress [91]. *ALA* acts as a cofactor for mitochondrial enzymes and promotes glucose metabolism by stimulating glucose uptake through the redistribution of GLUT-1 and GLUT-4 glucose transporters on cell membranes. ALA improves insulin sensitivity and reduces insulin and glucose levels [85]. Two studies in overweight/obese PCOS patients evaluated the effectiveness of 400 mg of *ALA* administered daily for 12 weeks. The results showed an improvement in insulin sensitivity and liver enzyme levels (AST and ALT) [90,92]. In addition, a recent study showed that *ALA* also improved hepatic insulin clearance. These findings suggested that *ALA* could be beneficial in enhancing the metabolic profile of patients with PCOS, thereby decreasing the risk of complications such as MASLD and diabetes [92].

Interestingly, a combination of *MYO* and *ALA* has been shown to further improve metabolic and hormonal parameters [85]. In adolescents, the combination of 400 mg *ALA* and 1000 mg *MYO* twice a day for three months, and then once a day for a further three months, improved both the metabolic profile and the inflammatory status by lowering the pro-inflammatory mediator high mobility group box 1 protein (HMGB1), and by reducing ovarian volumes and androgen precursors in serum [93].

Obese adult PCOS patients treated with *MYO* (2 g/day) and *ALA* (200 mg/di) for eight weeks showed decreased BMI, LH, LH/FSH ratio, androstenedione, and insulin levels, with the most significant results observed in obese insulin-resistant PCOS patients [94].

Another study showed a significant reduction in LH, LH/FSH ratio, and improved HOMA-IR index following a combined therapy of *MYO* (1 g) and *ALA* (400 mg) for at least 12 weeks [95].

The effects of *myoinositol* in obese/overweight patients with PCOS has been further compared to those of metformin, showing no differences between the two treatments on body composition, hormone profile, glucose, insulin metabolism, and adiponectin levels in PCOS patients [96].

One randomized clinical trial compared the effects of *myoinositol* and *resveratrol* supplementation versus a pharmacological treatment with metformin and pioglitazone. The combination of *myoinositol* and *resveratrol* proved to be more effective in improving body composition, metabolic balance, and the hormonal profile of women with obesity and PCOS [97].

*Folic acid* supplementation has been reported to improve inflammatory factors and biomarkers of oxidative stress in obese/overweight women with PCOS. A 5 mg/daily supplementation of *folic acid* for eight weeks improved homocysteine and hs-CRP levels, increased total antioxidant capacity and glutathione levels, and reduced malondialdehyde levels [98]. Another study that used the same dose and duration of supplementation showed in addition an improvement of the lipid profile [99].

In conclusion, a combined supplementation of *MYO* and *DCI* seems to be particularly effective in enhancing insulin sensitivity, hormonal balance, and ovarian function. Additionally, *ALA* supplementation, at a dosage of 400 mg/day, improves insulin sensitivity and is indicated in insulin-resistant PCOS subjects. *Curcumin*, at a dosage of 1000 mg/day, also improves insulin sensitivity, reduces hyperandrogenism, and improves lipid profiles. Finally, *berberine* (500 mg twice/day) and *resveratrol* (1000 mg/day) are effective in improving hormone profiles and insulin sensitivity in overweight/obese women with PCOS.

The main nutraceuticals studied in PCOS patients are summarized in Table 2.

## 6. Mental Health and Evidence for the Use of Nutraceutical Compounds

Obesity can cause not only physical comorbidities but can affect self-esteem, body image, lead to the development of psychopathology and psychosocial complications, and provoke even psychiatric disorders [8]. The most common mental health issues during childhood are anxiety and depression [100]. Stigmatization, poor body image, and low self-esteem can lead to depression. Along with poor dietary habits, lack of physical activity, and sleeping disorders, which also may lead to depression [101]. Biological mechanisms such as inflammation, impaired glucose metabolism, changes in the hypothalamic–pituitary–adrenocortical axis, and neuroendocrine mechanisms via leptin melanocortinergic-BDNF signaling that have been reported to be implicated in the development of depression [102] as well as genetic factors [103].

Lifestyle changes should be the primary treatment for obese individuals with mental health issues. Researchers reported that a short-term, mild calorie-restricted diet can have antidepressant effects in both animals and humans [104].

Previous studies have shown positive effects of nutraceuticals in treating psychological or psychiatric issues [18], but there is a notable lack of research involving obese subjects. Furthermore, the few limited studies focus exclusively on the adult population.

*Vitamin D* has been investigated as a potential therapy essential for normal brain development and function [105,106]. As previously explained, obese subjects have lower serum levels of *vitamin D*. *Vitamin D* deficiency can increase the risk of developing depression due to its ability to upregulate genes involved in the catecholamine pathway and increase the synthesis of monoamine neurotransmitters that promote neuroplasticity and have an immunomodulatory and anti-inflammatory role. Moreover, it is hypothesized that *vitamin D* has a protective role in reducing the negative effects of dopaminergic toxins, possibly by increasing glial cell-line-derived neurotrophic factors [107,108]. A meta-analysis reported that *vitamin D* supplementation of 4000 IU daily or more has been found beneficial for reducing depressive symptoms in adults [109], and 2000 IU daily for adolescents [110].

Current studies highlight the importance of *probiotic* supplements, as a high-calorie diet can lead to dysbiosis and affect emotional behavior in humans [111]. In vivo studies found that supplementation with *Lactobacillus rhamnosus* attenuated depressive symptoms in obese male mice [112]. A recent meta-analysis in adult patients has shown positive effects of *probiotics* on symptoms of anxiety, depression, and cognitive issues, especially in people with mild symptoms [113]. However, another meta-analysis did not support the use of *probiotics* to reduce symptoms of depression in children and adolescence [7]. Therefore, considering these heterogeneous results, large scale clinical studies are required.

Supplementation with *inulin* at a dose of 10 g/day for 8 weeks was rather ineffective on depression symptoms in obese women [114]. However, further studies are needed to evaluate the real efficacy of prebiotics and probiotics in mental disorders in obese subjects, particularly in pediatric patients.

*Zinc* supplementation (30 mg/day) in obese women has been shown to have positive effects on cognition, anxiety, and depression independent of any weight-loss intervention [115]. In fact, zinc has a neuroprotective, anti-inflammatory, and anti-oxidative action, as reported in some studies [115,116,117].

A single randomized clinical trial showed that *extra virgin olive oil* and *Brazilian diet* are able to improve symptoms of anxiety and depression in adults with severe obesity [118].

Therefore, there is currently little information on possible dietary supplements that might be useful for the treatment of mental health disorders in obese subjects. The few available studies concern adults only and cannot be considered entirely reliable. The samples analyzed were small, trials were performed for a limited period of time, and confounding factors were not evaluated. As a general indication, *vitamin D* supplementation seems to be once again advisable, if insufficient, and pre- and probiotics might be of help. Further studies are needed.

## 7. Conclusions

This review has highlighted current evidence on the use of nutraceuticals and bioactive compounds, within specific obesity-related complications and comorbidities, namely hypertension, PCOS, bone mineral density loss, and mental disorders.

Nutraceutical compounds exhibit a range of beneficial effects, including anti-inflammatory, antioxidant, insulin-sensitizing, and lipid-lowering properties. They have been shown to improve endothelial function, reduce vascular resistance, modulate gut microbiota, enhance glucose homeostasis and insulin secretion, inhibit osteoclastogenesis, and decrease levels of pro-osteoclastogenic cytokines.

Based on the current evidence, omega-3 fatty acids and vitamin D have demonstrated antihypertensive effects in adults, with some promising albeit limited data emerging in pediatric populations. Similarly, polyphenols, omega-6 fatty acids, and vitamin D may favor bone health, although evidence in children remains scarce and inconsistent, further research is required. For PCOS, polyphenols, probiotics, inositols, alpha-lipoic acid, and folic acid have shown positive effects on metabolic and hormonal parameters, especially in adults, with some evidence in adolescents suggesting promise. Regarding mental health, vitamin D, zinc, and probiotics may be neuroprotective, but more robust data are needed to guide their use in younger individuals.

Nutraceuticals should not be considered substitutes for diet and lifestyle modifications; they may function as adjuncts or alternatives when conventional therapies are not achievable. Most compounds offer the potential for preventive strategies that can be initiated early in life, particularly during childhood.

As this review was not a systematic review, some selection bias may be present. However, the literature available on nutraceutical interventions in pediatric patients is limited, and many findings were extrapolated from adult studies or preclinical models, which may not fully reflect pediatric ages. In addition, there is considerable heterogeneity among studies in terms of design, sample sizes, intervention types, dosages, duration, and outcome measures, making direct comparisons and generalizations challenging. The short duration of most pediatric trials also limits conclusions on long-term efficacy and safety of these compounds. Furthermore, the absence of standardized dosing and the variability in nutraceutical formulations complicate the interpretation of results. Finally, the clinical use of nutraceuticals in children with obesity is not yet supported by consensus guidelines, limiting their integration into routine practice.

Concluding, childhood obesity is a multifaceted condition that can lead to a broad spectrum of comorbidities, including metabolic and cardiovascular diseases (PCOS, impaired bone mineral density, and mental health disturbances). While lifestyle modifications remain the cornerstone of treatment, accumulating evidence highlights the potential role of nutraceutical compounds as complementary interventions in mitigating obesity-related complications.

The current evidence supports the use of specific nutraceuticals in improving blood pressure, hormonal balance, bone mineral density, and mental well-being in children and adolescents with obesity.

Although nutraceuticals may enhance or even partially substitute pharmacological approaches, especially when tailored to individual needs, their integration into clinical practice must be guided by stronger evidence. A personalized, evidence-based approach that combines nutraceuticals with lifestyle and dietary interventions holds potential for improving outcomes and preventing long-term complications in pediatric obesity.

Therefore, future research should prioritize large-scale, well-controlled pediatric trials that investigate optimal dosing, long-term safety, and efficacy of specific nutraceuticals for targeted comorbidities, with an emphasis on age- and sex-specific responses.

## Figures and Tables

**Table 1:** Summary of nutraceutical supplements that have been studied in children with obesity-related hypertension.

Nutraceutical	Dose	Sample Size	Positive Effects	Limitations	References
*Omega-3 fatty acids*	2.4 g/day for 1 month	39 patients (11–12 yr) overweight with SM	Reductions in both SBP and DBP, improved lipid profiles and glucose levels	Limited number of studies in children, longer-term safety and efficacy of the treatment in this population is required	[34]
	1.5 g/day for 16 weeks	78 male patients (13–15 yr) overweight	SBP was 3.8 ± 1.4 mmHg lower (*p* < 0.006) and DBP was 2.6 ± 1.1 mmHg lower (*p* < 0.01) in the receiving fish oil group compared with the control group		[35]
	130 mg DHA + 25 mg EPA associated with antioxidant vitamins for 3 months	65 obese (11.8 ± 3.5 yr) and 35 normal weight (11.6 ± 3.4 yr)	SBP was 133.2 ± 12.9 mmHg obese children before the treatment and 119.2 ± 12.2 mmHg after the treatment		[36]
	100 to 350 mg/day	300 obese children (fifth and sixth graders)	Omega-3 fatty acids induce changes BP in a dose-dependent manner		[37]
*Vitamin D (cholecalciferol)*	600 to 1000 or 2000 IU/day for 6 months	225 patients (10 to 18 yr) overweight and obese	Reduction of central-systolic, central-diastolic, and systemic-diastolic BP at 6 months is more effective in the 1000–2000 IU group	Conflicting results regarding effects on BP in pediatric population	[39]

**Table 2:** Summary of nutraceutical compounds that have been studied in patients with PCOS.

Nutraceutical	Dose, Administration Period	Sample Size (Age Range)	Positive Effects	Limitations	References
*Essential amino acids*	15 g/day, 4 weeks	21 patients (12–21 yr)	Improves AST and triglyceride levels	Single short-term study	[64]
*Probiotics*	1 capsule/day of 10 billion *CFUs* for 2 months then 2 capsules/day of 10 billion *CFUs* for 4 months 2 capsules/day of 1 billion *CFUs*, 12 weeks	104 patients, 52 receiving probiotics, 52 receiving placebo (18–40 yr) 60 patients, 30 receiving probiotics, 30 receiving placebo (18–45 yr)	Improves weight-loss, menstrual regularity, testosterone and insulin levels, LH/FSH ratio	Studies in adult patients only	[66,67]
*Green tea*	500 mg/twice a day, 12 weeks	60 patients, 30 receiving green tea, 30 receiving placebo (20–40 yr)	Improves body composition, insulin, and testosterone levels	Single short-term study in adult patients	[71]
*Curcumin*	500 mg/day, 12 weeks 500 mg/twice a day, 12 weeks 93.34 mg/day, 8 weeks	50 patients, 24 receiving curcumin, 26 receiving placebo (18–40 yr) 67 patients, 34 receiving curcumin, 33 receiving placebo (18–49 yr) 30 patients, 15 receiving curcumin, 15 receiving placebo (20–35 yr)	Improves glucose and insulin levels, insulin sensitivity, and lipid balance, lowers body weight, BMI, and hyperandrogenism	Studies in adult patients only. Conflicting results regarding hormone level improvement	[72,73,74]
*Quercetin*	1000 mg/day, 12 weeks	78 patients, 39 receiving quercetin, 39 receiving placebo (20–40 yr)	Lowers resistin, insulin, testosterone, and LH levels	Single short-term study in adult patients.	[75]
*Resveratrol*	1500 mg/day, 3 months 1000 mg/day, 3 months	30 patients, 15 receiving resveratrol, 15 receiving placebo (26.8 ± 1.1 yr) 78 patients, 39 receiving resveratrol, 39 receiving placebo (18–40 yr)	Improves menstrual regularity and hyperandrogenism	Low efficacy in hyperandrogenism at lower doses. Studies are in adult patients only	[76,77]
*Pomegranate juice*	2 litres of pomegranate juice + 20 g of insulin + 200 million *CFU*/g of lactobacillus a week, 8 weeks 45 mL/day, 8 weeks	86 patients, 22 receiving synbiotic pomegranate juice, 22 receiving pomegranate juice, 21 receiving synbiotic juice, 21 receiving placebo (15–48 yr) 42 patients, 21 receiving pomegranate juice, 21 receiving placebo (18–40 yr)	Improves insulin sensitivity and testosterone levels, decreases weight and waist circumference	Limited evidence in adolescents	[78,79]
*Carnitine, L-arginine, and N-acetylcysteine*	250 mg carnitine + 500 mg, L-arginine + 50 mg *N*-acetylcysteine/day, 24 weeks	45 patients (adults, age not specified)	Lowers insulin levels and HOMA-IR index, improves lipid parameters	Studies are in adult patients only. No changes in hormonal levels	[82]
*Berberine*	500 mg/twice a day, 6 months	50 patients (25.0 ± 3.5 yr)	Improves visceral adiposity, HOMA-IR, lipid profile and menstrual regularity, lower androgens	Single study in adult patients	[83]
*Inositols (Myo-inositol and D chiro-inositol)*	550 mg *MYO* + 13.8 mg *DCI*, twice a day, 6 months	50 patients, 26 receiving *MYO* + *DCI*, 24 receiing *MYO* (18–41 yr) 46 patients, 21 receiving *MYO* + *DCI* + folic acid, 25 receiving folic acid as placebo (23.0 ± 6.8 yr) 43 patients, 12 receiving *MYO* + *DCI* + diet, 10 receiving *MYO* + diet, 21 only diet (16–45 yr)	Improves ovulation and insulin sensitivity	Some studies are in small series	[85,87,88,89]
*Alpha-lipoic acid*	400 mg/day, 12 weeks	32 patients (24.5 ± 1.3 yr) 32 patients (adults, age not specified)	Improves insulin sensitivity, metabolic parameters, and liver enzyme levels	Studies in adult patients only	[90,92]
*Myo-inositol* + *Alpha-lipoic acid*	1000 mg *MYO* + 400 mg *ALA*/day for 3 months, then twice a day for 3 months 2000 mg *MYO* + 200 mg *ALA*/day, 8 weeks 1000 mg *MYO* + 400 mg *ALA*/day, 12 weeks	23 patients (17.22 ± 0.72 yr) 42 patients (adults, age not specified) 34 patients (26.4 ± 0.8 yr)	Improves metabolic parameters and inflammatory status. Lowers BMI, LH/FSH, androstenedione, and insulin levels	Limited sample size	[93,94,95]
*Folic acid*	5 mg/day, 8 weeks	69 patients, 23 receiving folic acid 5 mg/day, 23 receiving folic acid 1 mg/day, 23 receiving placebo (18–40 yr) 81 patients, 27 receiving folic acid 5 mg/day, 27 receiving folic acid 1 mg/day, 27 receiving placebo (18–40 yr)	Improves inflammatory factors, oxidative stress biomarkers, and lipid profile	Studies in adult patients only	[98,99]

Note. AST—aspartate aminotransferase, LH/FSH ratio—luteinizing hormone to follicle stimulating hormone ratio, BMI—Body mass index, HOMA-IR—Homeostatic Model Assessment for Insulin Resistance, LH—luteinizing hormone, MYO—Myo-inositol, DCI—D chiro-inositol, ALA—Alpha-lipoic acid.

## Data Availability

No new data were created or analyzed in this study.

## Abbreviations

The following abbreviations are used in this manuscript:

PCOS	Polycystic ovary syndrome
CVD	Cardiovascular disease
T2DM	type 2 diabetes mellitus
MASLD	metabolic dysfunction-associated steatotic liver disease
OSA	obstructive sleep apnea
BP	blood pressure
RAAS	renin-angiotensin-aldosterone system
AD	Adiponectin
DASH	Dietary Approaches to Stop Hypertension
PUFAs	polyunsaturated fatty acids
DHA	docosahexaenoic acid
EPA	eicosapentaenoic acid
NOS	nitric oxide synthase
Ca^2+^	calcium
BMI	body mass index
AAP	American Academy of Pediatrics
IU	international units
LH	luteinizing hormone
SHBG	sex hormone binding globulin
CFU	colony forming units
FSH	follicle-stimulating hormone
LDL	low density lipoprotein
HDL	high density lipoprotein
HOMA-IR	homeostasis model assessment—insulin resistance
MYO	myo-inositol
DCI	D chiro-inositol
ALA	alpha-lipoic acid
GLUT-1	glucose transporter 1
GLUT-4	glucose transporter 4
AST	aspartate aminotransferase
ALT	alanine aminotransferase
MASLD	Metabolic Dysfunction-Associated Steatotic Liver Disease
HMGB1	high mobility group box 1
hs-CRP	high sensitivity-C reactive protein
RANK	receptor activator of nuclear factor k B
RANKL	receptor activator of nuclear factor k B ligand
GTP	green tea polyphenols
IGF-1	insulin growth factor 1
PTH	parathyroid hormone
BDNF	brain-derived neurotrophic factor
